# *In situ* green oxidation synthesis of Ti^3+^ and N self-doped SrTiO_*x*_N_*y*_ nanoparticles with enhanced photocatalytic activity under visible light

**DOI:** 10.1039/c7ra13523h

**Published:** 2018-02-14

**Authors:** Jiandong Liu, Xiaohong Ma, Lina Yang, Xingliang Liu, Aixia Han, Haitang Lv, Chao Zhang, Shiai Xu

**Affiliations:** School of Chemical Engineering, Qinghai University Xining 810016 China zhangchaoqhu@126.com saxu@163.com +86-971-5310427 +86-971-5310427; Shanghai Key Laboratory of Advanced Polymeric Materials, Key Laboratory for Ultrafine Materials of Ministry of Education, School of Materials Science and Engineering, East China University of Science and Technology Shanghai 200237 China

## Abstract

A simple *in situ* green oxidation synthesis route was developed to prepare Ti^3+^ and N self-doped SrTiO_*x*_N_*y*_ nanoparticles using TiN and H_2_O_2_ as precursors. X-ray diffraction (XRD), scanning electron microscopy (SEM) and high-resolution transmission electron microscopy (HRTEM) were used to characterize the crystallinity, structure and morphology. X-ray photoelectron spectroscopy (XPS) tests confirmed the presence of Ti^3+^ and N in the prepared SrTiO_*x*_N_*y*_ nanoparticles. The resultant nanoparticles were shown to have strong absorption from 400 to 800 nm using UV-vis diffuse reflectance spectroscopy (UV-vis DRS). The formation mechanism of the Ti^3+^ and N self-doped SrTiO_*x*_N_*y*_ nanoparticles was also discussed. Under visible light irradiation, the obtained Ti^3+^ and N self-doped samples showed higher photocatalytic activity for the degradation of the model wastewater, methylene blue (MB) solution. The most active sample T-130-Vac, obtained at 130 °C under vacuum, showed a 9.5-fold enhancement in the visible light decomposition of MB in comparison to the commercial catalyst nano-SrTiO_3_. The sample also showed a relatively high cycling stability for photocatalytic activity.

## Abstract 2

A simple *in situ* green oxidation synthesis route was developed to prepare Ti^3+^ and N self-doped SrTiO_*x*_N_*y*_ nanoparticles using TiN and H_2_O_2_ as precursors.

## Graphical abstract

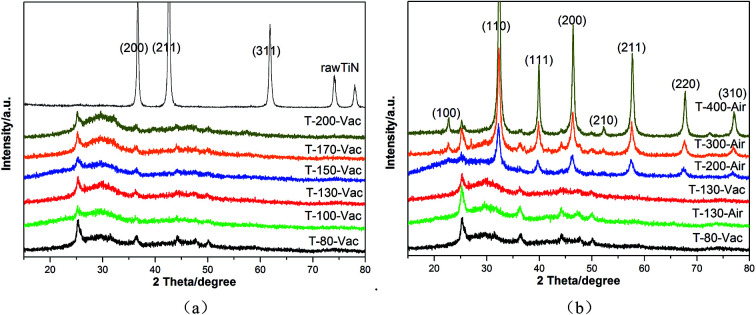

## 1. Introduction

The perovskite SrTiO_3_, one of the most promising photocatalysts, has gained much increased attention in recent years due to its high chemical stability and good photoelectric performance in the reduction of organic pollutants and the production of hydrogen.^1–5^ However, due to the wide band gap (about 3.2 eV), SrTiO_3_ only shows activity in the ultraviolet (UV) region.^6^ To enhance the photocatalytic efficiency, current efforts are focused on narrowing the band gap of SrTiO_3_ to harvest visible light. An effective approach to solve this challenge is to dope SrTiO_3_ structures with various cations or anions.^7–11^ As mono-doping may cause dopant-induced charge recombination and/or traps, co-doping has been adopted to overcome these limitations in SrTiO_3_.^12–17^ However, some significant limitations posed by low doping concentrations or dopant-induced thermal instability still need to be resolved.^18^

Recently, Ti^3+^ and oxygen vacancy doped SrTiO_3_ has attracted much attention. It is reported that the self-doping of oxygen vacancies is important and this kind of oxygen vacancy in perovskite oxides would cause critical impact on their optical properties.^19^ However, some theoretical calculations have suggested that the concentration of Ti^3+^ must be high enough to induce higher oxygen deficiency which may cause linear vacancy ordering with defect–defect interactions in the perovskite. Then, the higher oxygen deficiency induces a new in-gap band which may enhance visible light absorption,^19^ which has also been well confirmed in Ti^3+^ and oxygen vacancy doped TiO_2_.^20^ It is reported that visible light absorption and the photocatalytic activities of the catalysts under visible light irradiation can be improved using this kind of strategy.^21,22^

To date, however, only a few methods are capable of producing Ti^3+^ and/or oxygen vacancy doped SrTiO_3_. Hou *et al.* reported a facile routine to synthesize Ti^3+^ self-doped SrTiO_3_/TiO_2_ heterostructures with substantially enhanced visible light activity using an evacuated two-zone furnace of 800 °C (molten AL) and 450 °C (SrTiO_3_/TiO_2_) for 6 h under 5 × 10^−4^ Pa pressure reduction.^23^ Ye *et al.* obtained a self-doped SrTiO_3_ photocatalyst with enhanced visible light activity using a carbon-free combustion method involving TiN and Sr(NO_3_)_2_ in air. In this process, TiN and Sr(NO_3_)_2_ were dissolved in a HNO_3_ and 30% H_2_O_2_ mixed solution, then the obtained precursors were heated at 1200 °C for 20 h in air to get the final yellow powder sample.^19^ From the reported method, it can be seen that these methods are mostly carried out under harsh, costly, and often complicated conditions. Due to the harsh environments used for preparing N doped TiO_2_ or Ti^3+^ doped TiO_2_, it is still a great challenge to develop facile synthetic routes and it seems that the compounds could not be realized simultaneously by current methods.^24^ The same challenges also remain for the development of facile synthetic routes for N and Ti^3+^ self-doped SrTiO_3_. Besides, there are almost no reports about the preparation of Ti^3+^ or N self-doped amorphous or crystal SrTiO_3_ and its photocatalytic activity. Thus, there is a pressing need to develop a facile synthetic strategy to achieve a high concentration of Ti^3+^ doping throughout the bulk crystal or amorphous SrTiO_3_ matrix with improved thermal and chemical stabilities.

We have demonstrated herein an *in situ* green oxidation method for the preparation of a highly active yet stable Ti^3+^ or N self-doped amorphous SrTiO_*x*_N_*y*_ containing a high concentration of Ti^3+^ and N embedded inside the matrix. In contrast to the current reduction-based method or the high temperature combustion method, we obtained a green oxidation-based method under low temperatures to produce high concentration doping of Ti^3+^ and N throughout the bulk and surface of the amorphous photocatalysts. TiN, hydrogen peroxide (H_2_O_2_) and Sr(OH)_2_·8H_2_O were used as precursors. TiN is a widely used and abundant industrial material in wear and corrosion-resistant coatings for various tools, machine parts or consumer goods and is highly stable in air and water at ambient or even high temperatures.^25^ H_2_O_2_ was selected as the oxidation agent, which is environmentally friendly and highly selective and removes the chance of unnecessary impurities in the final product.^26^ As the oxidation state of the titanium species in TiN is +3, a controllable oxidative to convert some of the Ti^3+^ species to Ti^4+^ species can be easily realized using differing amounts of H_2_O_2_. Besides, Sr(OH)_2_·8H_2_O as the alkaline substance can easily react with the acid oxidation precursor which can largely decrease the reaction temperature. The formed amorphous or crystal Ti^3+^ and N self-doped SrTiO_*x*_N_*y*_ powder showed a high visible light absorption and enhanced photocatalytic activities under visible light. Such a solution-based oxidative method with careful design and selection of precursor could contribute to the scalable and low-cost production of a highly efficient Ti^3+^ and N self-doped amorphous or crystal SrTiO_*x*_N_*y*_ photocatalyst within the existing industrial infrastructure.

## 2. Experimental

### 2.1. Sample preparation

In this paper, the Ti^3+^ and N self-doped amorphous or crystal SrTiO_*x*_N_*y*_ nanoparticles were prepared by a simple *in situ* green oxidation method using TiN (99.9%, 20 nm, Shanghai Chao Wei Nano Technology Co., Ltd.) and Sr(OH)_2_·8H_2_O (99.5%, AR, Aldrich) as precursors. In the typical preparation procedure, 0.60 g TiN powder was dispersed in 10 mL distilled water and 20 mL H_2_O_2_ solution (30 wt%, AR, Sinopharm Chemical Reagent Co., Ltd) under stirring in a 250 mL beaker. The mixture was kept continuously stirring for 120 min at room temperature. After that, 2.60 g Sr(OH)_2_·8H_2_O (control molar ratio of Ti : Sr = 1 : 1) was added into the mixed solution under magnetic stirring for another 120 min. Then, we obtained a light blue gel and the resulting mixture was dried at 80 °C in a vacuum for 15 hours, and then further heated at various temperatures and atmospheres under vacuum or air for 4 hours. Commercial SrTiO_3_ nanoparticles (NPs) (99.5%, 100 nm, Macklin) were used as the reference. The samples heated at different temperatures and atmospheres were designated *T*–*T*_x_–*E*, where *T*_x_ indicates the heating temperature (°C) and *E* indicates the heating atmosphere (vacuum (Vac) or air atmosphere (Air)).

### 2.2. Characterization

X-ray powder diffraction (XRD) analysis was carried out using a Rigaku D/MAX 2500X X-ray powder diffractometer with Cu Kα radiation. The morphology was observed using a Gemini 500/300 scanning electron microscope (SEM). Higher resolution transmission electron microscopy (HRTEM) was carried out using a transmission electron microscope (JEOL, JEM-2100F, Japan) operated at 200 kV. The X-ray photochemical spectra (XPS) were obtained on a Thermo SCIENTIFIC ESCALAB 250 equipped with Al Kα alpha radiation. Ultraviolet visible diffuse reflection spectra (UV-vis DRS) were recorded on an Agilent Cary 5000 UV-vis-NIR spectrophotometer in the range of 200–800 nm at room temperature.

### 2.3. Photocatalytic activity

Photocatalytic activity was evaluated by analyzing the degradation of methylene blue (MB) dye under visible light irradiation at room temperature. The visible light source was provided by a 300 W Xe lamp (PLS-SXE300, Beijing AuLight Co. Ltd.) with an ultraviolet cutoff filter (*λ* > 400 nm). In a typical experiment, 0.10 g of sample was dispersed into 100 mL of 10 ppm MB solution under constant stirring in a quartz vessel. The adsorption–desorption equilibrium was achieved by stirring the mixture in a dark environment for 30 min and then irradiating the mixture by visible light. During photocatalytic experiments, approximately 5 mL of suspension was taken out for detection after high speed centrifugation every 10 min. The UV-visible absorption spectra of the supernatant solutions were analyzed using a UV-visible spectrometer (Shimadzu UV-2550) in the range from 400 to 800 nm to monitor the characteristic absorption peak of MB (664 nm).

## 3. Results and discussion

### 3.1. Photocatalyst characterization

**Fig. 1:**
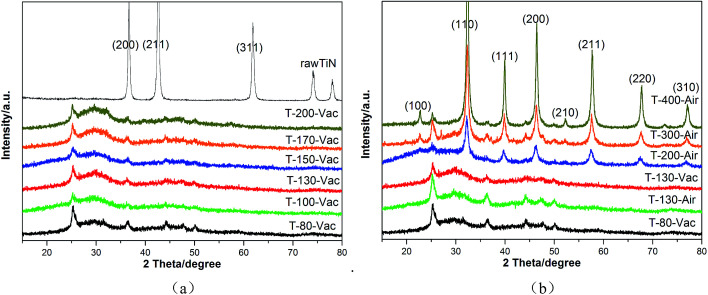
XRD patterns of raw material and gel after heating at various temperatures and atmospheres under vacuum (a) or air (b) for 4 hours.

Powder X-ray diffraction (XRD) analysis was used to investigate the crystal structure of the raw material (TiN), intermediate (the gel after desiccation but before heating treatment), and the samples after heating treatment under vacuum or air atmospheres at various temperatures (Fig. 1). From Fig. 1(a), it can be seen that the gel obtained by oven-drying under vacuum at 80 °C for 15 hours tended to be an amorphous powder. The typical diffraction peaks of the raw material TiN (JCPDS card no. 65-0715) can nearly not be observed in the figure, which shows the complete conversion of the starting powder, TiN, to amorphous SrTiO_*x*_N_*y*_. With the increase in the heating temperature, the powder remained amorphous even when the temperature was increased up to 200 °C, which shows its high heat resistance. The gel treated with different temperatures under air atmosphere was chosen for comparison. As can be seen from Fig. 1(b), with the heating treatment temperature increase from 130 °C to 400 °C under air atmosphere, the pattern exhibits characteristics from amorphous to good crystallization. The amorphous powder tended to crystallize for the T-200-Air sample treated under 200 °C in air. When the heating temperature reached 400 °C, the pattern of T-400-Air exhibited characteristic diffraction peaks matching the (110), (111), (200), (211), (220) and (310) planes of strontium titanate (JCPDS card no. 35-0734).

**Fig. 2:**
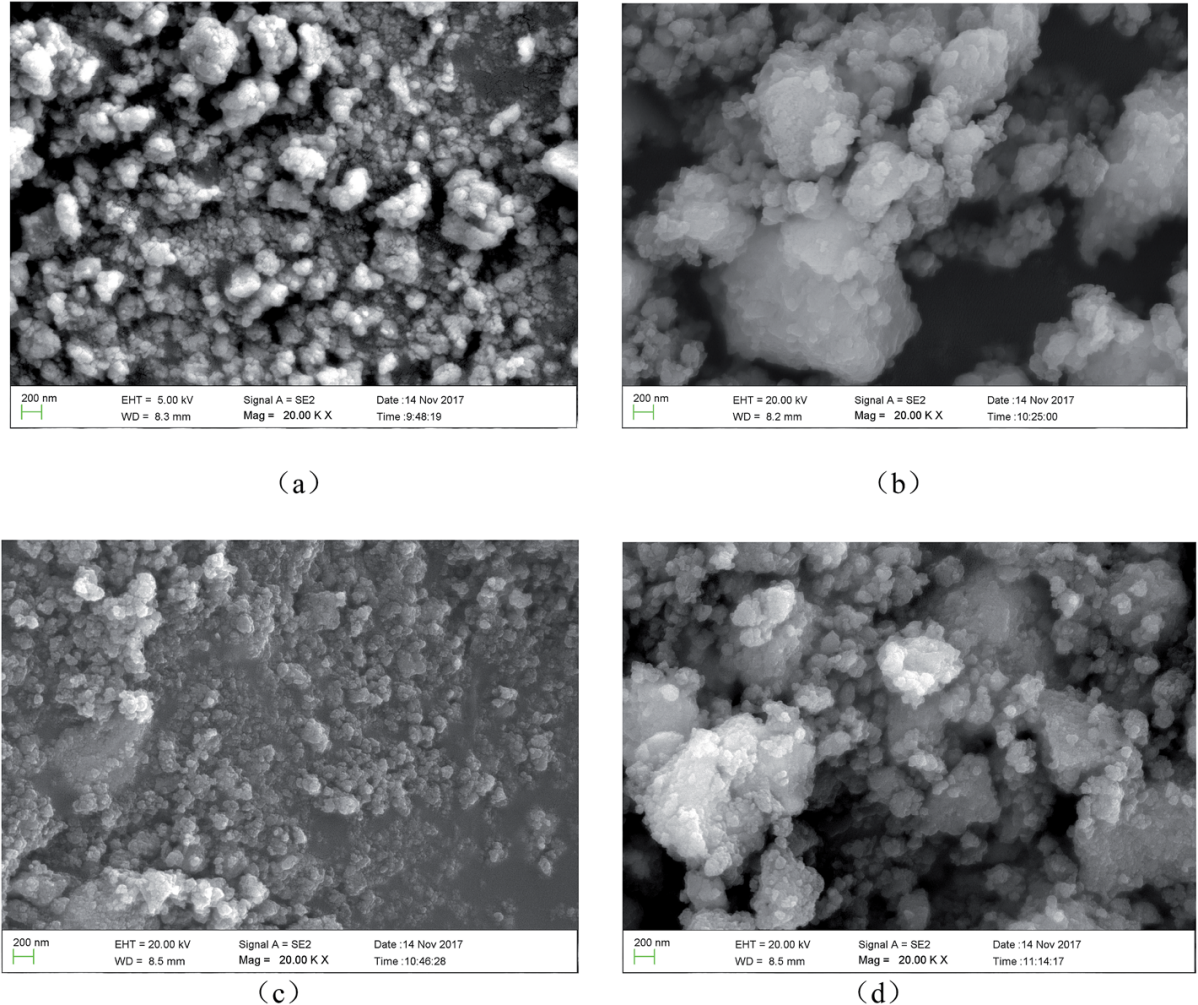
SEM images of Ti^3+^ and N self-doped SrTiO_*x*_N_*y*_ samples heated at various temperatures and atmospheres: (a) T-130-Vac, (b) T-200-Vac, (c) T-200-Air and (d) T-300-Air.

**Fig. 3:**
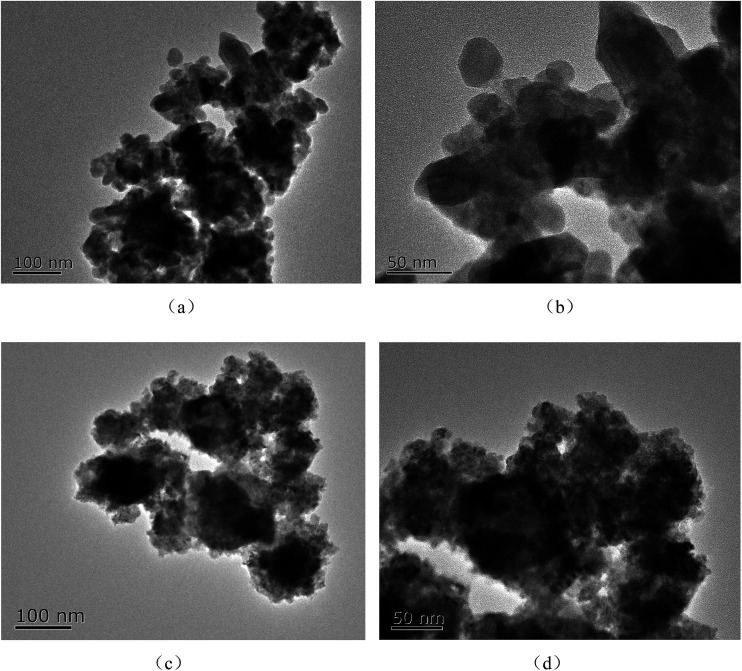
TEM images of Ti^3+^ and N self-doped SrTiO_*x*_N_*y*_ samples heated at various temperatures and atmospheres: (a and b) T-200-Vac heated at 130 °C for 4 h under vacuum, and (c and d) T-200-Air heated at 200 °C for 4 h in air.

The morphology of samples was observed using SEM. Fig. 2 shows the Ti^3+^ and N self-doped SrTiO_*x*_N_*y*_ samples heated at various temperatures and atmospheres. The average size of the T-130-Vac nanoparticles was approximately 50 nm and quite uniform as shown in Fig. 2(a). When treated at higher temperatures, the nanoparticles tended to aggregate. The particle size of T-200-Vac was about 100 to 200 nm (Fig. 2(a)). The same trend can also be observed for T-200-Air and T-300-Air as shown in Fig. 2(c and d), which means that treating at relatively lower temperatures or under vacuum is beneficial while ensuring high photocatalytic efficiency. Fig. 3 shows the TEM images of the T-200-Vac and T-200-Air samples. From the TEM images we can further confirm that the nanoparticulate T-200-Vac (∼100 nm) can be prepared and the aggregation trend is also found in T-200-Air (100–200 nm).

**Fig. 4:**
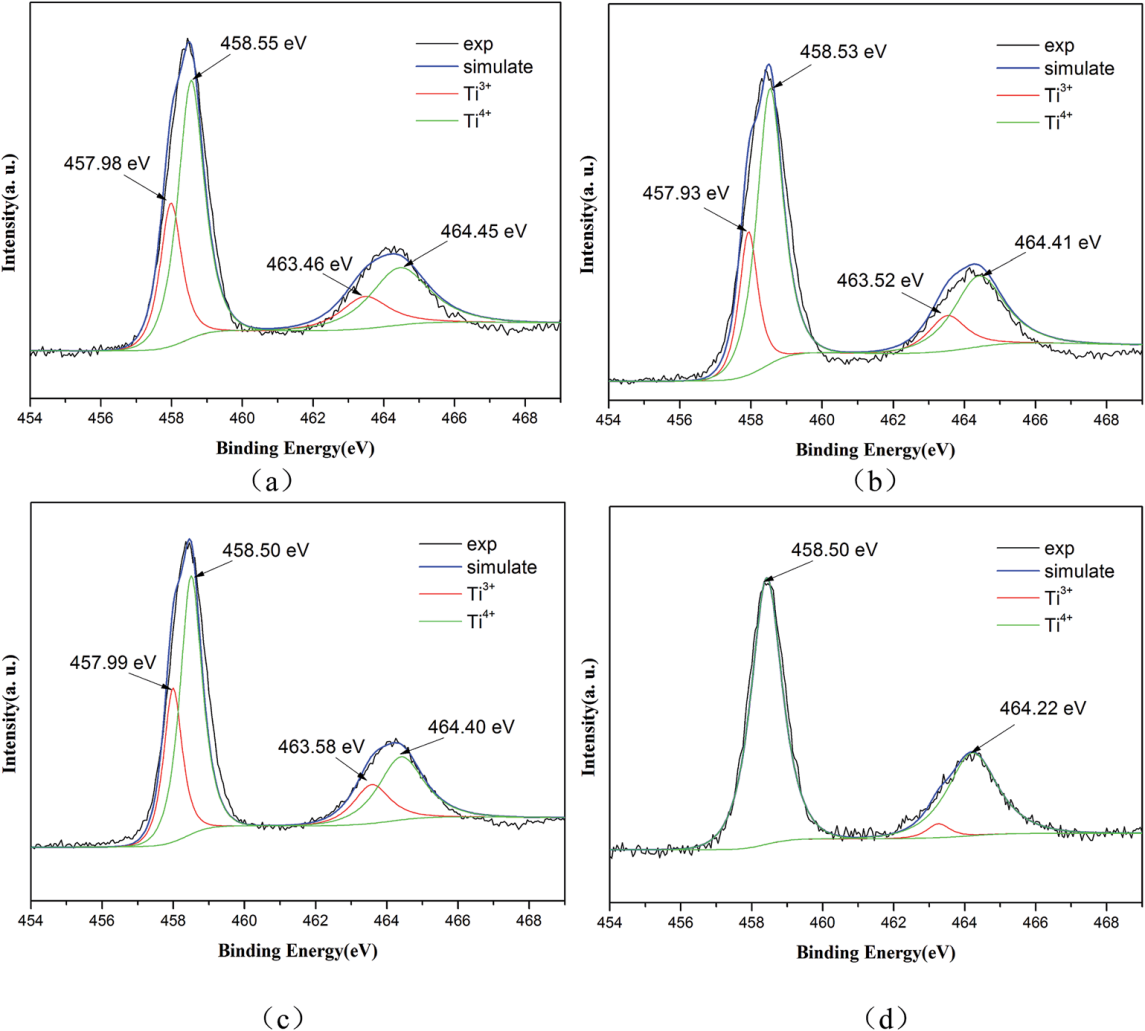
High-resolution XPS spectra of Ti 2p for Ti^3+^ and N self-doped SrTiO_*x*_N_*y*_ samples heated at various temperatures and atmospheres: (a) T-130-Vac, (b) T-200-Vac, (c) T-200-Air and (d) T-300-Air.

XPS measurements were carried out to investigate the presence and chemical states of Ti and N in the samples. The high-resolution XPS spectrum of Ti 2p (Fig. 4(a)) shows two peaks at the binding energies 458.50 eV and 464.40 eV, which can be ascribed to Ti 2p_3/2_ and Ti 2p_1/2_. Additionally, the Ti 2p peaks are well de-convoluted into four peaks as Ti^3+^ 2p_3/2_ (457.98 eV), Ti^4+^ 2p_3/2_ (458.55 eV), Ti^3+^ 2p_1/2_ (463.46 eV) and Ti^4+^ 2p_1/2_ (464.45 eV), which clearly demonstrates the existence of Ti^3+^ and Ti^4+^ ions.^24,26^ The similar binding energy peaks of Ti^3+^ and Ti^4+^ can also be seen in Fig. 4(b and c), showing samples T-200-Vac and T-200-Air. However, only two obvious peaks at 458.50 eV and 464.22 eV can be observed, which can be assigned to Ti^4+^ 2p_3/2_ and Ti^4+^ 2p_1/2_. The typical peak of Ti^3+^ has disappeared in Fig. 4(d), which shows the reduced Ti^3+^ content in the sample T-300-Air, which was treated at a higher temperature in air.

**Fig. 5:**
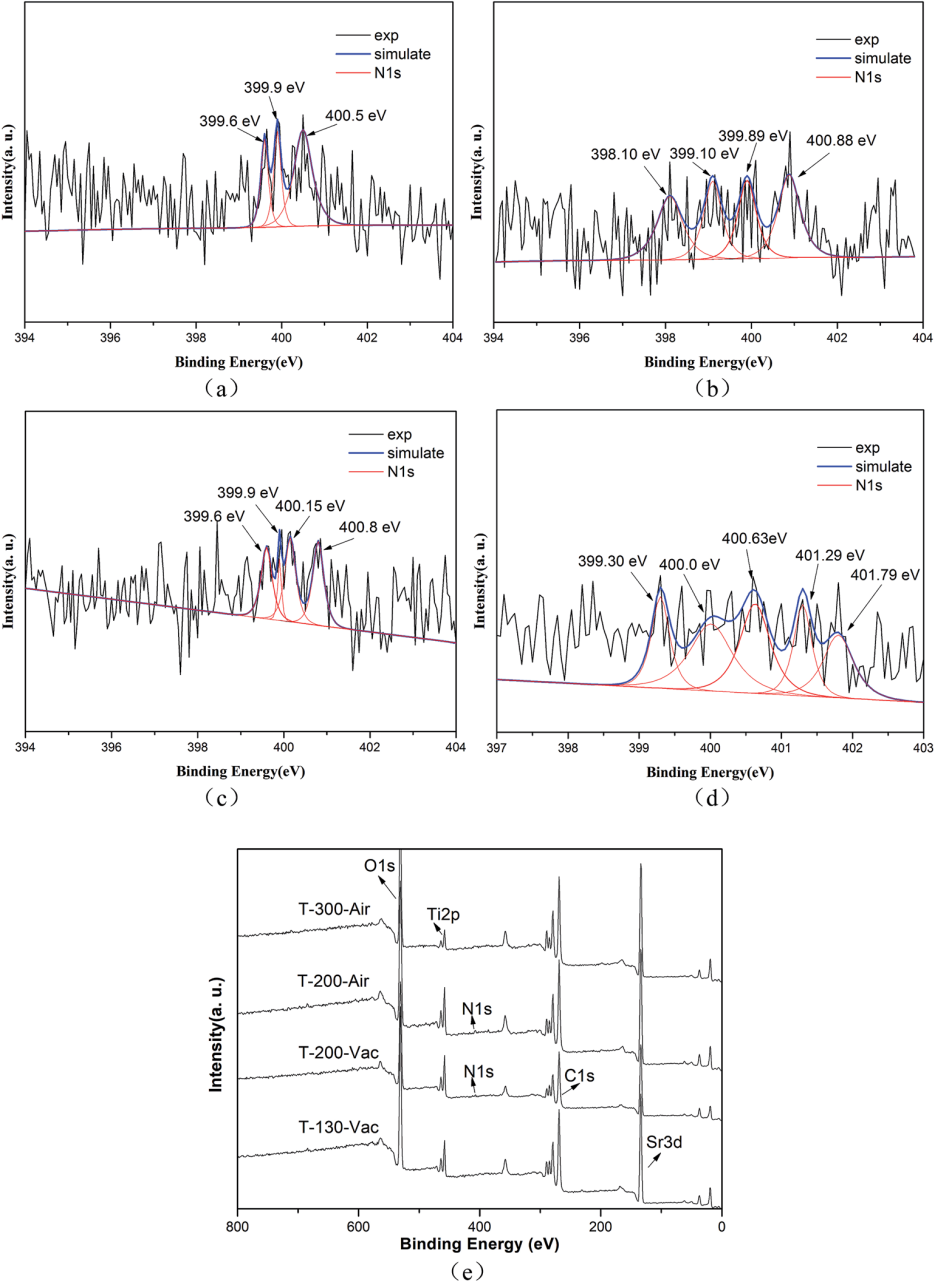
The high-resolution XPS spectra of N 1s for Ti^3+^ and N self-doped SrTiO_*x*_N_*y*_ samples heated at various temperatures and atmospheres: (a) T-130-Vac, (b) T-200-Vac, (c) T-200-Air, (d) T-300-Air and (e) the whole XPS spectra.

The N 1s XPS spectra are shown in Fig. 5 and we can see that the peaks of N 1s are broad and asymmetric, which means that there are more than two kinds of N chemical state. Correspondingly, three peaks at 399.6 eV, 399.9 eV and 400.5 eV can be clearly observed, implying the existence of various types of N state (as shown in Fig. 5(a)). The major peaks at 399.6 eV and 399.9 eV are assigned to substitutional N in the form of O–Ti–N. Meanwhile, the minor peak at 400.5 eV can be attributed to interstitial N in the environment of Ti–O–N in the T-130-Vac sample.^27^ Similar results are also obtained from the XPS N 1s spectra for the T-200-Vac and T-200-Air samples (as shown in Fig. 5(b and c)). However, there are plenty of small peaks of N 1s for the T-300-Air sample, mainly located at 400.63 eV, 401.29 eV and 401.79 eV, which shows the oxidized nitrogen in the sample treated at a higher temperature in air (as shown in Fig. 5(d)).^20^ From the whole XPS spectra (as shown in Fig. 5(e)), it can be concluded that the Ti^3+^ and N self-doped SrTiO_*x*_N_*y*_ samples were successfully obtained by the *in situ* green oxidation synthesis method reported herein.

### 3.2. UV-vis analysis of the catalysts

**Fig. 6:**
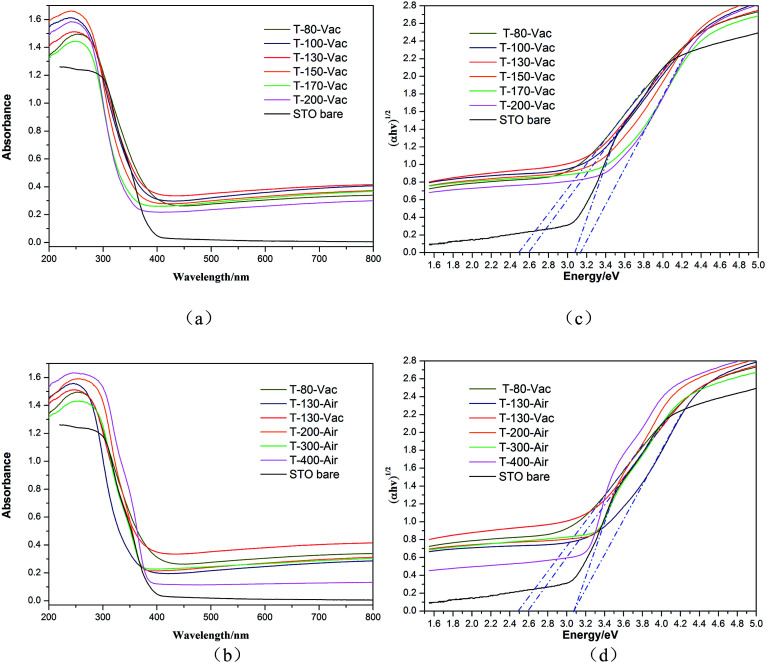
UV-vis DRS absorbance spectra of pure SrTiO_3_ and Ti^3+^ self-doped SrTiO_*x*_N_*y*_ samples heated at various temperatures and atmospheres under vacuum (a) or air (b), and the relationship between (*αhν*)^1/2^ and the photon energy of samples under vacuum (c) or air (d).

The optical properties of the Ti^3+^ and N self-doped SrTiO_*x*_N_*y*_ powder samples were analyzed by UV-vis diffuse reflectance spectra, as is shown in Fig. 6. The pure SrTiO_3_ and the samples treated under air atmosphere were also analyzed for comparison. It is clear that the obtained amorphous or crystal SrTiO_*x*_N_*y*_ powders under vacuum treated at various temperatures from 100 °C to 200 °C exhibit stronger broad absorption bands between 400 and 800 nm compared with pure SrTiO_3_, covering nearly the whole visible range (Fig. 6(a)). The amorphous SrTiO_*x*_N_*y*_ powder under vacuum exhibits strong broad absorption in visible light which is also thoroughly consistent with the light blue or blue colour of the samples. The UV-vis diffuse reflectance spectra of the precursor gel treated in air atmosphere for the obtained amorphous or crystal SrTiO_*x*_N_*y*_ powder treated with temperatures from 130 °C to 400 °C are shown in Fig. 6(b). With an increase in the treated temperature in air, the absorption of the sample in the visible region is weaker than that of the sample under vacuum which means that the atmosphere and temperature used are key factors and should be controlled carefully.

The band gap values were calculated from the UV-vis absorption spectra according to the following eqn (1):

1 *αhν* = *A*(*hν* − *E*_g_)^*n*/2^

where *α*, *hν*, *A*, and *E*_g_ are the absorption coefficient, the photon energy, the proportionality constant, and the band gap, respectively. *n* is equal to 1 or 4 depending on the direct or indirect transition (*n* = 4 for SrTiO_3_).^28,29^ The band gap energy *E*_g_ was calculated by extrapolating a straight line to the abscissa axis. Fig. 6(c and d) shows the plot of (*αhν*)^1/2^*versus* the photon energy (*hν*) for SrTiO_3_. The band gaps of pure SrTiO_3_ and the precursor gels treated under vacuum (T-100-Vac, T-130-Vac, T-150-Vac, T-170-Vac and T-200-Vac) are estimated as 3.12, 2.6, 2.6, 2.95, 3.1, and 3.11 eV, respectively. For comparison, the band gaps of the precursor gels treated under air atmosphere (T-130-Air, T-200-Air, T-300-Air and T-400-Air) were also calculated as 3.09, 3.1, 3.02 and 3.11 eV, respectively. It is clearly shown that the band gap increases with the temperature treatment of the precursor gel from low to high under vacuum or air atmosphere. Additionally, the band gap of the samples treated under vacuum is much smaller than that of the samples treated in air atmosphere, especially for T-130-Vac and T-130-Air, which means that we may obtain a new blue amorphous Ti^3+^ and N self-doped SrTiO_*x*_N_*y*_ powder under low temperatures in vacuum with high visible light absorption.

### 3.3. Photocatalytic activity

The photocatalytic activity of the Ti^3+^ and N self-doped SrTiO_*x*_N_*y*_ powder samples was evaluated using the degradation of MB, which is often used as a test model pollutant in semiconductor photocatalysis.

**Fig. 7:**
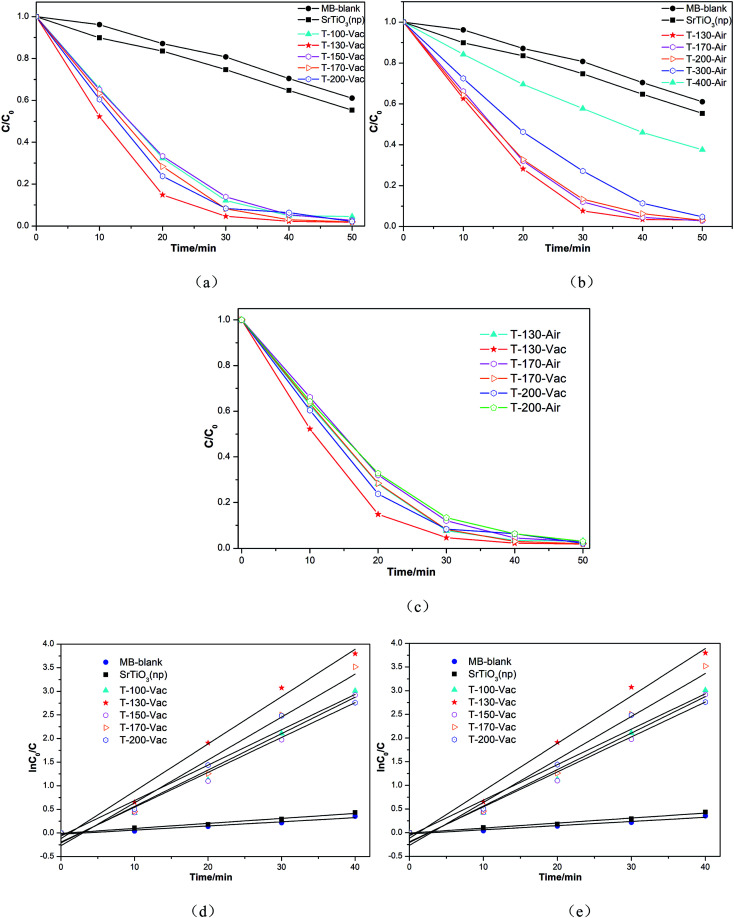
The photodegradation of MB solutions using commercial nano-SrTiO_3_ (NPs) and Ti^3+^ and N self-doped SrTiO_*x*_N_*y*_ as photocatalysts under visible light irradiation in neutral suspension. (a) Ti^3+^ and N self-doped SrTiO_*x*_N_*y*_ treated under vacuum, (b) Ti^3+^ and N self-doped SrTiO_*x*_N_*y*_ treated in air, (c) the comparison between the Ti^3+^ and N self-doped SrTiO_*x*_N_*y*_ treated under vacuum and the Ti^3+^ and N self-doped SrTiO_*x*_N_*y*_ treated in air and (d) variation in the normalized ln(*C*_0_/*C*) of the MB concentration as a function of irradiation time.

Fig. 7 shows the photodegradation ability of our obtained samples on MB solutions, heated at various temperatures and atmospheres under vacuum or in air, in comparison to that of nano-SrTiO_3_ (NPs). Under visible light illumination, the MB solutions containing the Ti^3+^ and N self-doped SrTiO_*x*_N_*y*_ powder samples underwent significant degradation and became nearly transparent within 30 min (as shown in Fig. 7(a)). It should be noted that the sample obtained at 130 °C for 4 h under vacuum shows the highest photocatalytic oxidation effect on MB. With an increase in the treated temperature under vacuum or in air atmosphere, the degradation efficiency tended to decrease and the sample obtained at 400 °C for 4 h under air atmosphere showed the lowest efficiency, although it was still higher than that of commercial nano-SrTiO_3_ (NPs) (as shown in Fig. 7(b)). From Fig. 7(c), we can determine that the degradation efficiency only changes a little with the samples treated at temperatures from 130 °C to 200 °C under vacuum or in air atmosphere, which means that our samples have a relatively high heat resistance, and the fact that the amorphous samples contain Ti^3+^ and N may be the key factor.

**Fig. 8:**
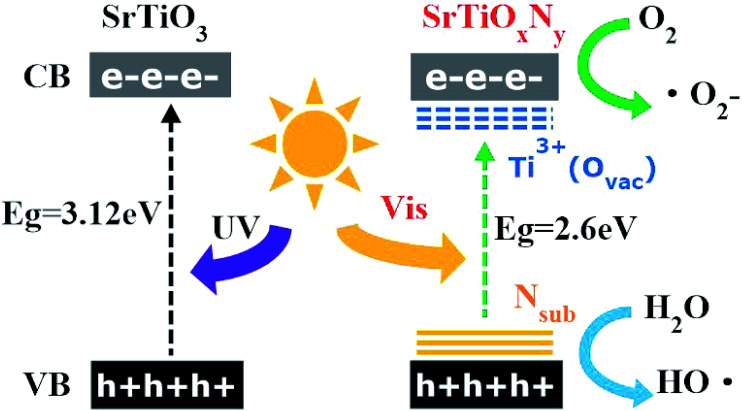
Schematic electronic band structure of SrTiO_3_ and SrTiO_*x*_N_*y*_ (Ti^3+^ (O_Vac_), N_sub_, *E*_g_, UV, vis denoted Ti^3+^ self-doping (oxygen vacancy), substituted nitrogen, band gap, UV light and visible light).

Additionally, we also calculated the reaction rate constants for the obtained photocatalyst degradation of MB by plotting ln(*C*_0_/*C*) *versus* the irradiation time interval with different samples in 40 min under visible light irradiation. The pseudo first-order rate constant *k* could be obtained from the linear time dependences of ln(*C*_0_/*C*), as shown in Fig. 7(d and e). The most active sample, T-130-Vac (obtained at 130 °C under vacuum), showed a 9.5-fold enhancement in the visible light decomposition of MB in comparison to the commercial catalyst nano-SrTiO_3_ (NPs). The high visible light photocatalytic activity of the obtained Ti^3+^ and N self-doped SrTiO_*x*_N_*y*_ is reasonable, as the donor level is induced by nitrogen doping upon the valence band (VB) inside the bandgap of SrTiO_*x*_N_*y*_, while Ti^3+^ self-doping introduces an impurity level just under the conduction band (CB).^22^ Then the CB of Ti^3+^ and N self-doped SrTiO_*x*_N_*y*_ is shifted towards the VB due to the Ti^3+^ self-doping and the VB maximum moves upwards due to the N-doping, which narrows the band gap and ensures the high visible light photocatalytic activity,^30^ as illustrated in Fig. 8.

**Fig. 9:**
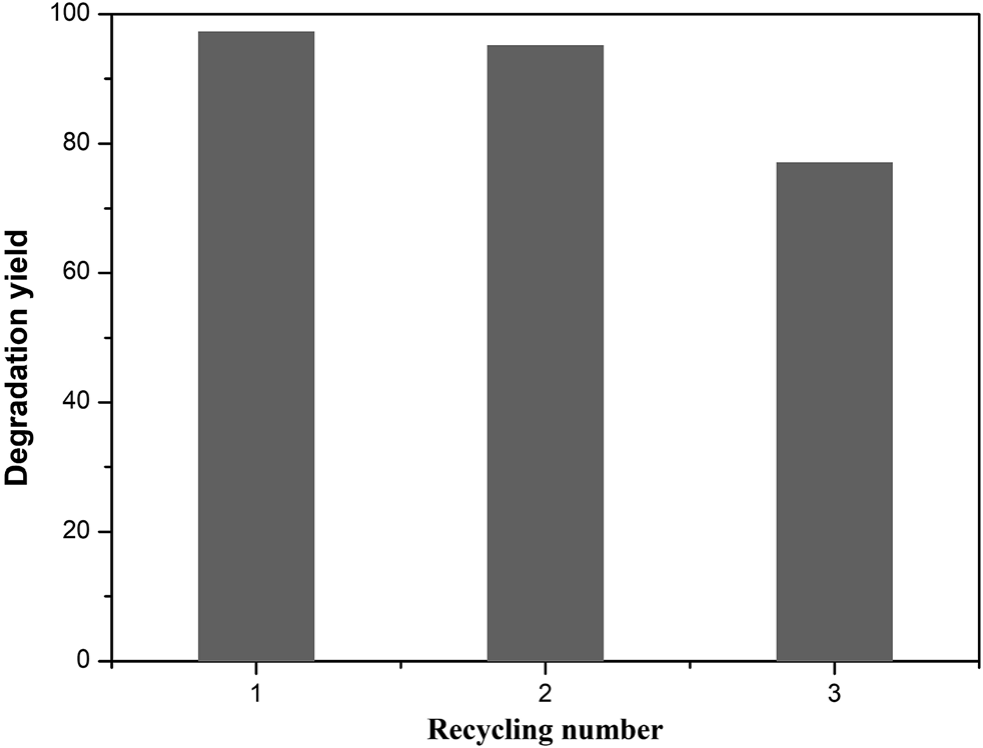
Recycling test results for the T-130-Vac sample.

Cycling stability tests of the T-130-Vac sample under the same conditions were also conducted and showed excellent photocatalytic activity for the degradation of MB solution, as shown in Fig. 9. It was found that ∼75% degradation yield was retained after 3 catalytic cycles for the sample synthesized at 130 °C under vacuum, which means that the relative stability of the obtained sample is acceptable, considering the simple preparation process and the potential value of easy-to-scale applications for the efficient photocatalytic removal of water waste.

### 3.4. Formation mechanism

**Fig. 10:**
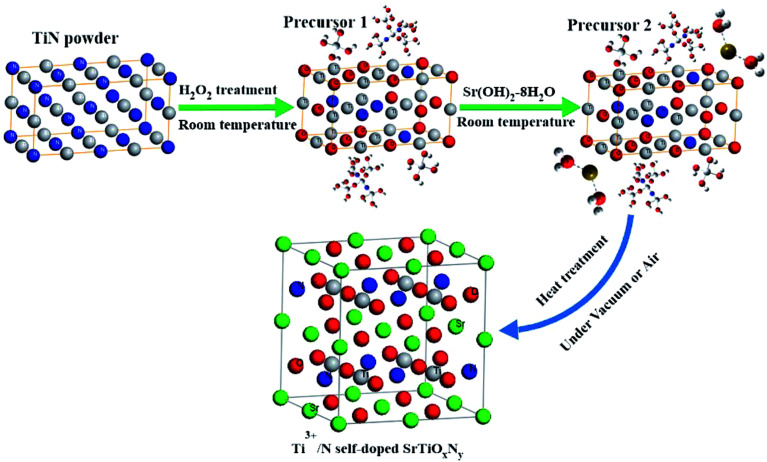
Schematic of the formation mechanisms for the Ti^3+^ and N self-doped SrTiO_*x*_N_*y*_ nanoparticles, precursor 1: [–N–Ti–O–]_*n*_–Ti–(OH)_*x*_–*m*H_2_O and precursor 2: Sr_*x*_[–N–Ti–O–/–O–Ti–O–]_*y*_.

Based on the obtained results above, a possible formation mechanism for the Ti^3+^ and N self-doped amorphous SrTiO_*x*_N_*y*_ nanoparticles is proposed, as shown in Fig. 10. At the initial stage of the reaction, the surfaces of the TiN powders were oxidized by H_2_O_2_. A surface layer with a complex chemical composition was formed on the TiN particles. This kind of process may be the same as TiH_2_ oxidized by H_2_O_2_ (ref. 31) or TiN oxidized under an electrochemical environment.^25^ This means that the formed surface layer was a mixture of various of titanium oxynitride/oxides and oxohydrides ([–N–Ti–O–/–O–Ti–O–]_*n*_–Ti–(OH)_*x*_–*m*H_2_O, Fig. 10 precursor 1). From the experimental process we also see that at this stage, the initial black colored sol changes to an army green sticky sol, and the reaction rate changes from slow to very fast at the end. This kind of phenomenon is consistent with the supposed cross-linked [–O–Ti–O–]_*n*_–Ti–(OH)_*x*_–*m*H_2_O matrix formed by reacting TiH_2_ powders with H_2_O_2_.^18^ After the temperature of precursor 1 fell back to room temperature, a certain amount of Sr(OH)_2_·8H_2_O (control molar ratio of Ti : Sr = 1 : 1) was added to precursor 1. We could then quickly obtain a light blue gel precursor 2 and the main composition may be non-stoichiometric Sr_*x*_ [–N–Ti–O–/–O–Ti–O–]_*y*_. This is reasonable as precursor 1 [–N–Ti–O–/–O–Ti–O–]_*n*_–Ti–(OH)_*x*_–*m*H_2_O has weak acidity (pH = 3–4) while Sr(OH)_2_ is a strong alkaline (pH = 13–14) and this kind of reaction can easily happen, which is also consistent with the observed, quickly obtained, light blue gel precursor 2 after Sr(OH)_2_·8H_2_O was added into the army green sticky sol precursor 1 by our experiment. In the dry process under vacuum and the differing heat treatment conditions, the resulting precursor 2 Sr_*x*_ [–N–Ti–O–/–O–Ti–O–]_*y*_ formed amorphous Ti^3+^ and N self-doped SrTiO_*x*_N_*y*_ powder eventually. Moreover, the concentrations of Ti^3+^ and N could be controlled by the amount of H_2_O_2_ used and the heat temperature or atmosphere. With an increase in heating treatment temperature from 130 °C to 400 °C under air atmosphere, the pattern exhibits characteristics including amorphous behaviour and good crystallization, and the pattern exhibits a characteristic diffraction peak matching the peak of strontium titanate.

## 4. Conclusion

In conclusion, we have developed a simple and economical *in situ* green oxidation synthesis route to obtain Ti^3+^ and N self-doped SrTiO_*x*_N_*y*_ nanoparticles using TiN and H_2_O_2_ as precursors. The resulting Ti^3+^ and N self-doped SrTiO_*x*_N_*y*_ exhibits strong visible light absorption and photocatalytic activity in the degradation of model organic water waste. Besides, the obtained Ti^3+^ and N self-doped sample shows good stability in air, even up to 200 °C, and can be repeatedly used without a significant reduction in degradation activity.

## Conflicts of interest

There are no conflicts to declare.

## Supplementary Material
